# Influence of thyroid autoimmunity at various clinical stages of hypothyroidism on the risk of miscarriage before 20 weeks of gestation

**DOI:** 10.1007/s42000-023-00474-2

**Published:** 2023-08-22

**Authors:** Mohammed Ali Gameil, Rehab Elsayed Marzouk, Ahmed Hassan Elsebaie, Ahmed Abd EL-Hakim Arafat, Mohammed Ibrahim Abd El-Ghany

**Affiliations:** 1 Endocrinology Unit, Internal Medicine Department, Faculty of Medicine, Mansoura University, Dakahlia, Mansoura, Egypt; 2 Medical Biochemistry Department, Faculty of Medicine, Helwan University, Helwan, Cairo, Egypt; 3 Clinical Pathology Department, Faculty of Medicine, Mansoura University, Dakahlia, Mansoura, Egypt

**Keywords:** Miscarriage, Hypothyroidism, Thyroid autoantibodies, Pregnancy

## Abstract

**Purpose:**

We aimed to clarify the influence of thyroid autoantibodies at various clinical stages of hypothyroidism on the risk of pregnancy loss before 20 weeks of gestation.

**Methods:**

We enrolled 230 pregnant women with a history of recurrent miscarriage. Detailed clinical history, physical examination, and laboratory testing of thyroid function, antithyroid peroxidase (anti-TPO), and antithyroglobulin (anti-TG) were applied among all participants.

**Results:**

Coexisting overt hypothyroidism and positive thyroid autoantibodies quadrupled the risk of miscarriage in women before 20 weeks of gestation (OR 4.04, 95% CI = 2.08–7.96, *P* < 0.001). Women with subclinical hypothyroidism (OR 1.44, 95% CI = 0.81–2.57, *P* = 0.132,) or who were euthyroid (OR 1.53, 95% CI = 0.86–2.73, *P* = 0.094) showed a non-significant risk of miscarriage even with positive thyroid autoantibodies. Thyroid-stimulating hormone (TSH) was positively correlated with the number of miscarriages rather than anti-TPO (*P* < 0.001 and 0.209, respectively).

**Conclusion:**

Coexistence of overt hypothyroidism and thyroid autoimmunity was the only significant driver of pregnancy loss before 20 weeks of gestation.

## Introduction

Thyroid dysfunction and thyroid autoimmunity are well known to be associated with adverse gestational outcomes. Pregnancy loss is a frequent complication in women with evident thyroid autoimmunity [1, 2]. Worldwide, hypothyroidism is the most common type of thyroid dysfunction with the potential to become heterogeneous. Autoimmune thyroid disease is well-recognized as a principal cause of hypothyroidism. It is prevalent in about 5–15% of females of reproductive age and is associated with adverse pregnancy outcomes [3]. Disruption of the immune system through production of autoantibodies may induce specified or generalized structural and/or functional derangement, with the thyroid gland potentially being damaged as a targeted organ of specific autoantibodies. Although many types of thyroid autoantibodies have been discovered, the most common types are those which are directed against thyroid peroxidase and thyroglobulin molecules [4–6]. The pathogenesis underlying thyroid autoimmunity-related adverse pregnancy outcomes is still controversial. Twig et al. [7] and Yamamoto et al. [8] attributed miscarriage in women with thyroid autoimmunity to TSH-dependent and TSH-independent effects. Overproduction of interferon-γ, reduced interleukin 10, endometrial T-cell dysfunction, and a surge of cytotoxic natural killer cells in the uterus may account for the TSH-independent effect. Negro et al. [9] attributed pregnancy loss associated with thyroid autoimmunity to progressive lack of sufficient thyroid hormone to cope with normal gestational changes. Other researchers have described thyroid autoantibodies as an anti-conception factor that often induces miscarriage, particularly in advanced maternal age [10, 11]. The American Society of Reproductive Medicine (ASRM) Practice Committee Report defined recurrent miscarriages as the loss of two or more consecutive pregnancies: it is a phenomenon that occurs in about 1–2% of couples who wish to conceive. Miscarriage is the spontaneous loss of a fetus before 20 weeks of gestation. The rate of miscarriage varies greatly, with approximately 30% of miscarriages occurring before the recognition of pregnancy and 10–15% of pregnancy loss being reported before the 8th week and about 3% of miscarriages between the 8th and the 20th week of gestation [12]. Although approximately half of the cases of miscarriage are reported to be of unknown etiology, recurrent miscarriage is particularly prevalent among women with thyroid disorders, uncontrolled hyperglycemia, hyperprolactinemia, antiphospholipid syndrome, genetic anomalies, and maternal pelvic anomalies [13, 14].

The impact of thyroid autoimmunity throughout the different stages of hypothyroidism on the risk of miscarriage before 20 weeks of gestation is still unclear. We aimed to pinpoint specific variations in the influence of thyroid autoimmunity on the risk of pregnancy loss in women with a prior history of miscarriage at various clinical stages of hypothyroidism.

## Methods

Our study is an observational descriptive study that included 230 pregnant women aged 18–35 years with a singleton pregnancy. The cases included pregnant women before 20 weeks of gestation with a prior history of pregnancy loss. They were selected from the obstetric outpatient department of Mansoura University Hospital, Mansoura, Dakahlia, Egypt, and comprised women who were making regular visits for antenatal care during the period from June 2021 to June 2022. All participants were interviewed for a detailed medical history and underwent a clinical examination and laboratory testing of thyroid function and thyroid autoantibodies. The clinical history included personal and family history of thyroid disorders as well as of other autoimmune diseases such as systemic lupus erythematous, celiac disease, type 1 diabetes mellitus, and other endocrine disorders, while recording also took place of any history of thyroid surgery, therapeutic neck irradiation, current or previous administration of antithyroid drugs, levothyroxine replacement therapy, or radioiodine therapy, and a detailed obstetric history including past history of miscarriage, preterm labor, pre-eclampsia, and other gestational complications. We excluded women with hyperthyroidism, thyroid malignancy, other endocrine disorders, other known autoimmune diseases, cardiovascular diseases, epilepsy, severe anemia, systemic arterial hypertension, decompensated liver, renal, cardiac, or pulmonary diseases, antiphospholipid syndrome, and patients with a history of prior thyroid surgery, therapeutic neck irradiation, or administration of medications that may alter thyroid function, such as amiodarone, corticosteroids, lithium, antipsychotic, levothyroxine, or antithyroid medications for at least 12 months before enrollment. We excluded participants with anatomical abnormalities in the cervix and uterus, women with a prior history of congenital anomalies of fetus, and smokers.

After at least 8 h overnight fasting, a 10 ml venous blood sample was withdrawn from each woman with a vacutainer. The samples were centrifuged at 112 g for 10 min and the sera were analyzed for thyroid-stimulating hormone (TSH), free triiodothyronine (FT3), free tetraiodothyronine (FT4), antithyroid peroxidase antibody (anti-TPO), and antithyroglobulin antibody (anti-TG) by electrochemiluminescence immunoassay (Coobas e411, Roche Diagnostics, Germany). The normal TSH range is 0.4–4.2 mIU/l, for FT4 it is 12.0–22.0 pmol/l, for FT3 3.95–6.8 pmol/l, for anti-TPO <35 IU/ml, and for anti-TG <115 IU/ml. A value of anti-TPO over 35 IU/ml was considered positive for the presence of anti-TPO. The intra-assay coefficients of variations (CV) for TSH, anti-TPO, FT4, FT3, and anti-TG were 8.1–6.6, 3.25–10.98, 2.4–11.9, 4.2–5.7, and 4.1–6.2%, respectively. The inter-assay CVs were 5.9–9.3, 6.01–10.81, 10.2–13.1, 4.5–6.8, and 4.5–6.9%, respectively. Owing to the lack of trimester-specific reference ranges for evaluation of thyroid function among pregnant women in Egypt, the guidelines of the American Thyroid Association (ATA) for the diagnosis and management of thyroid diseases during pregnancy and postpartum were implemented [15]. Participants were subdivided into three categories, namely, euthyroid, subclinical hypothyroid, and overt hypothyroid. Participants with serum TSH more than 10.0 mIU/l, irrespective of FT4 level, and serum TSH more than 2.5 mIU/l in the first trimester or more than 3 mIU/l in the second trimester with reduced FT4 level were considered to have overt hypothyroidism. Subclinical hypothyroidism was defined as a serum TSH level between 2.5 and 10 mIU/l with a normal FT4 level.

###  Statistical analysis

Statistical analysis was carried out using SPSS statistical package (SPSS Inc) version17 (SPSS Inc, Chicago, IL, USA). Qualitative variables are presented as absolute and relative (%) frequencies, while quantitative variables are presented as mean ± SD or median (min-max), according to their distribution. Student’s *t*-test was used for the normally distributed data, while the Mann-Whitney *U*-test was used for non-normally distributed data. Linear correlation with Spearman’s correlation coefficient was implemented to determine a significant correlation between thyroid autoantibodies and various parameters. Multivariable logistic regression analysis was applied to assign the odds ratio of the association between miscarriage and different clinical thyroid entities in participants with positive anti-TPO. A *P*-value ≤ 0.05 indicated statistical significance level.

## Results

Table 1 presents the demographic characteristics of the women with a history of miscarriage with or without anti-TPO. Positive anti-TPO was prevalent in 71 of the women (30.8%) along with significantly elevated TSH and lower FT3 and FT4 (*P* < 0.001 for all) than comparators without positive anti-TPO.

**Table 1:** Demographic characteristics of women with a history of miscarriage with and without positive anti-TPO

Variable	Positive anti-TPO (*N* = 71)	Negative anti-TPO (*N* = 159)	*P* value
Age (years)	27.2 ± 3.21	27.6 ± 3.62	0.371
Gestational age (week)	9.38 ± 3.28	9.98 ± 3.75	0.240
Number of miscarriages *N* (%)			
Two	25 (35.21%)	56 (35.22%)	
Three	37 (52.11%)	86 (54.10%)	
>Four	9 (12.68%)	17 (10.68%)	
TSH (uIU/ml)	6.19 ± 5.65	2.81 ± 2.66	<0.001*
FT3 (pmol/l)	4.67 ± 0.823	5.18 ± 0.831	<0.001*
FT4 (pmol/l)	14.2 ± 2.99	15.6 ± 2.43	<0.001*
Anti-TPO (IU/ml)	160 (36–276)	28 (6–34)	<0.001*
Anti- TG (IU/ml)	135 (10–250)	32 (10–45)	<0.001*

Quantitative variables are presented as mean ±SD or median (min, max)

Test of significance: (*t*): independent samples *t*-test, Mann-Whitney *U* test

*TSH* thyroid-stimulating hormone, *FT3* free tri-iodothyronine, *FT4* free tetra-iodothyronine, *anti*-*TPO* antithyroid peroxidase antibody, *anti*-*TG* anti-thyroglobulin antibody

Table 2 illustrates variations of thyroid function tests and thyroid autoantibodies in women with a history of miscarriage at various clinical stages of hypothyroidism. Anti-TPO and anti-TG were significantly elevated in 28 (17.83%) of 157 women in euthyroid state (*P* < 0.001 for both). Out of 51 women with subclinical hypothyroidism, 29 (56.86%) showed significantly elevated anti-TPO and anti-TG (*P* = 0.005 and *P* <0.001), respectively. In 22 women with overt hypothyroidism, 14 (63.64%) exhibited a significant elevation of anti-TPO and anti-TG (*P* = 0.041, *P* = 0.008), respectively. Therefore, in women with a prior history of miscarriage, the prevalence of positive anti-TPO was observed to be accompanied by an increasingly deteriorating hypothyroid state, specifically, 17.83% in euthyroid, 56.86% in subclinical hypothyroid, and 63.64% in overt hypothyroid cases.

**Table 2:** Thyroid function tests and thyroid autoantibodies at various clinical stages of hypothyroidism among women with a history of miscarriage

Variable	Positive anti-TPO	Negative anti-TPO	*P* value
Euthyroid pregnant women (*N* = 157) *N* (%)	28 (17.83%)	129 (82.17)	
TSH (uIU/ml)	1.81 ± 0.739	1.72 ± 0.588	0.492
FT3 (pmol/l)	5.16 ± 0.766	5.37 ± 0.757	0.201
FT4 (pmol/l)	16.5 ± 2.12	16.1 ± 2.20	0.450
Anti-TPO (IU/ml)	152 (36–275)	25 (6–30)	<0.001*
Anti- TG (IU/ml)	146 (10–249)	33 (10–35)	<0.001*
Subclinical hypothyroid pregnant women (*N* = 51)	29 (56.86%)	22 (43.14%)	
TSH (uIU/ml)	6.77 ± 1.78	6.24 ± 1.68	0.291
FT3 (pmol/l)	4.59 ± 0.640	4.59 ± 0.514	0.963
FT4 (pmol/l)	13.9 ± 1.91	14.4 ± 1.80	0.350
Anti-TPO (IU/ml)	142 (36–276)	28 (18–34)	0.005*
Anti- TG (IU/ml)	125 (10–210)	30 (27–45)	<0.001*
Clinical hypothyroid pregnant women (*N* = 22)	14 (63.64%)	8 (36.36%)	
TSH (uIU/ml)	13.8 ± 7.75	10.9 ± 3.29	0.340
FT3 (pmol/l)	3.86 ± 0.543	3.80 ± 0.615	0.830
FT4 (pmol/l)	10.2 ± 1.49	10.9 ± 0.756	0.230
Anti-TPO (IU/ml)	195(38–272)	29(25–32)	0.041*
Anti- TG(IU/ml)	140(30–250)	30(26–33)	0.008*

Quantitative variables are presented as mean ±SD or median (min, max)

Test of significance: (*t*): independent samples *t*-test. Mann-Whitney *U* test

*TSH* Thyroid-stimulating hormone, *FT3* free tri-iodothyronine, *FT4* free tetra-iodothyronine, *anti*-*TPO* anti-thyroid peroxidase antibody, *anti-TG* anti-thyroglobulin antibody

Table 3 depicts the correlation between thyroid autoantibodies and other studied parameters among women with a history of miscarriage. A significant positive correlation was observed between TSH with anti-TPO (*P* = 0.022) and the number of miscarriages (*P* < 0.001). Interestingly, anti-TPO showed a non-significant correlation with the number of miscarriages (*P* = 0.209). Overall, TSH was inversely correlated with FT4 and FT3 (*P* < 0.001) in women with positive anti-TPO.

**Table 3:** Correlation between thyroid autoantibodies and other studied parameters in women with a history of miscarriage

Variable		TSH	Anti-TPO	Anti-TG	FT4	FT3	Age	Gest. age	NMIS
TSH	*r*	1	.269^*^	.102	−.639^**^	−.540^**^	−.121	.031	.492^**^
	*P*		.03	.378	<0.001	<0.001	.352	.724	<0.001
Anti-TPO	*r*	.269^*^	1	.301^*^	.035	.006	.010	−.121	.125
	*P*	.03		.024	.885	.954	.931	.347	.239
Anti-TG	*r*	.102	.301^*^	1	.010	−.013	−.092	.018	.125
	*P*	.378	.024		.934	.918	.491	.884	.304
FT4	*r*	−.639^**^	.035	.010	1	.619^**^	.082	−.117	−.185
	*P*	<0.001	.885	.934		<0.001	.496	.381	.135
FT3	*r*	−.540^**^	.006	−.013	.619^**^	1	.103	−.134	−.210
	*P*	<0.001	.954	.918	<0.001		.392	.266	.079
Age	*r*	−.121	.010	−.092	.082	.103	1	.066	−.012
	*P*	.352	.931	.491	.496	.392		.586	.924
Gest. age	*r*	.031	−.121	.018	−.117	−.134	.066	1	.118
	*P*	.724	.347	.884	.381	.266	.586		.328
NMIS	*r*	.492^**^	.125	.125	−.185	−.210	−.012	.118	1
	*P*	<0.001	.239	.304	.135	.079	.924	.328	

Test of significance: Spearman’s correlation coefficient

*Gest. age* gestational age, *NMIS* number of miscarriages

*Correlation is significant at the 0.05 level (2-tailed); **Correlation is significant at the 0.01 level (2-tailed)

Table 4 shows the adjusted odds ratio from multivariate logistic regression for the association between positive anti-TPO and the risk of miscarriage before 20 weeks of gestation at different clinical stages of hypothyroidism. Overt hypothyroidism significantly quadrupled the risk of pregnancy loss (OR 4.04, 95% CI = 2.08–7.96, *P* < 0.001). Meanwhile, subclinical hypothyroidism (OR 1.44, 95% CI = 0.81–2.57, *P* = 0.132) or the euthyroid state (OR 1.53, 95% CI = 0.86–2.73, *P* = 0.094) did not achieve a statistically significant level of association between positive anti-TPO and the risk of miscarriage.

**Table 4:** Adjusted odds ratio from multivariate logistic regression for the association between positive anti-TPO and the risk of miscarriage before 20 weeks of gestation at different clinical stages of hypothyroidism

Variable	Odds ratio (95% CI)	*P* value
Euthyroid pregnant women (*N* = 28)	1.53 (0.86–2.73)	0.094
Subclinical hypothyroid pregnant women (*N* = 29)	1.44 (0.81–2.57)	0.132
Overt hypothyroid pregnant women (*N* = 14)	4.04 (2.08–7.96)	<0.001*

## Discussion

Our study included 230 pregnant women with a history of miscarriages, with a mean age of 27.5 years and a mean gestational age of 9.8 weeks. We noted significantly elevated TSH along with lower FT4 and FT3 in women with positive anti-TPO than comparators with negative anti-TPO. Our results are in line with those of Chen and Hu [16] who reported higher TSH in women with miscarriage and thyroid autoantibodies than in women without thyroid autoimmunity. The prevalence of positive anti-TPO was accompanied by a steadily advancing clinical deterioration of hypothyroidism (17.83% in euthyroid, 56.86% in subclinical hypothyroid, and 63.64% in overt hypothyroid women). Our results are consistent with those of Dhillon-Smith et al. [17] who noted progressively increased prevalence of anti-TPO with worsened hypothyroidism, specifically, 17% in euthyroid, 40% in subclinical hypothyroidism, and 69% in clinical hypothyroidism. This finding clearly underlines the role of thyroid autoimmunity in deteriorating thyroid function. Bliddal et al. [18] reported a dose-dependent relationship between anti-TPO and TSH level in pregnant women and thus emphasized the importance of the anti-TPO assay as an initial marker for suspected thyroid autoimmunity in pregnancy.

Among our participants, the prevalence of thyroid autoimmunity was 30.86% among women with a history of miscarriage, this being in accord with Lata et al. [19] who reported positive anti-TPO, namely, approximately 31% among women with a prior history of miscarriage.

Interestingly, our participants exhibited a significant positive correlation between TSH, and not anti-TPO, with the number of miscarriages. Moreover, the risk of miscarriage was approximately quadrupled in women with coexisting overt hypothyroidism and positive anti-TPO. Our results are in line with those published by Ahmed et al. [20] who reported a significant negative association between maternal overt hypothyroidism and poor fetal outcomes. On the other hand, women with subclinical hypothyroidism or who were euthyroid did not show a significantly increased risk of pregnancy loss before 20 weeks of gestation even though exhibiting evident positive anti-TPO. Clinical thyroid status, in particular overt hypothyroidism, was therefore observed to outweigh thyroid autoantibodies for the risk of pregnancy loss before 20 weeks of gestation. This finding may explain the significant positive correlation between TSH rather than anti-TPO with the number of miscarriages. By contrast, other researchers attributed adverse gestational outcomes and pregnancy loss to thyroid autoimmunity irrespective of thyroid function status [21, 22]. Lata et al. [19] and Prummel et al. [23] have previously reported a double risk of miscarriage among women with positive anti-TPO. Moreover, Boogaard et al. [3] reported a tripled risk of miscarriage in early pregnancy which was associated with thyroid autoantibodies. Variations in study design, methodology, population ethnicity, and cutoff values of anti-TPO, TSH, and FT4 levels among the aforementioned studies could explain this inconsistency [24].

In the current study, women with subclinical hypothyroidism with thyroid autoantibodies were not at a significant risk of pregnancy loss (OR 1.44, *P* = 0.132). Our results are hence in agreement with those of Dong et al. [25] who conducted a systematic review and meta-analysis and found no significant risk of miscarriage in women with subclinical hypothyroidism and thyroid autoimmunity. They nevertheless emphasized the need for screening and treatment of overt hypothyroidism in pregnant women with a history of miscarriage. In addition, Plowden et al. [26] also reported no evidence of a significant correlation between subclinical hypothyroidism and thyroid autoantibodies with undesirable pregnancy outcomes. Meanwhile, Sitoris et al. [27] reported increased incidence of adverse pregnancy outcomes, including pre-eclampsia and low birth weight, rather than pregnancy loss in women with subclinical hypothyroidism and thyroid autoimmunity. Negro et al. [9] and Rao et al. [28] recommended administration of levothyroxine in women with thyroid autoantibodies regardless of thyroid function with the aim of minimizing the risk of miscarriage; however, recently, Negro [29] found no significant benefits of administration of levothyroxine in women with subclinical hypothyroidism with or without thyroid autoimmunity for the purpose of decreasing the risk of adverse outcomes. Our results are not in agreement with those published by Liu et al. [30] and Beneventi et al. [31] who observed an increased risk of miscarriage in women with subclinical hypothyroidism (OR 3.40, *P* = 0.002), which increased significantly in women with positive thyroid autoantibodies (OR 9.56, *P* <0.001). Variations in study population, design, and methodology may explain this inconsistency.

In the current study, euthyroid women who had positive anti-TPO were not at a significant risk of pregnancy loss (OR 1.53, *P* = 0.094). Our results are in accord with those of Yuan et al. [32] who reported a non-significant effect of thyroid autoimmunity in women with normal thyroid function on adverse gestational outcomes. On the contrary, Negro et al. [33] and Chen et al. [34] noted an increased incidence of adverse gestational outcomes, such as preterm labor, respiratory distress, and premature rupture of membranes, rather than pregnancy loss in women with thyroid autoimmunity and a euthyroid state. In the current study, the correlation between maternal age and thyroid autoimmunity lacked statistical significance, this being in agreement with Rajput et al. [35] and Stricker et al. [36] who detected no significant association between maternal age and thyroid autoimmunity. Meanwhile, Zhou et al. [37] reported advanced maternal age in pregnant women with thyroid dysfunction.

The current study has a number of limitations, namely, the single ethnicity population, the single-center study design, the relatively small sample size of individual subgroups, the lack of population-based trimester-specific reference range of thyroid function tests in our region, and lastly, a lack of tools to detect the molecular, histological, and pathological alterations between study groups. On the other hand, our inclusion of participants at different clinical stages of hypothyroidism with and without thyroid autoantibodies revealed statistical variances, data which are likely to support health care providers in their clinical practice. Larger multicenter studies with multiple ethnic populations are warranted in the future.

## Conclusion

Concomitant overt hypothyroidism and positive thyroid autoantibodies confer the highest risk of pregnancy loss before 20 weeks of gestation. Clinical thyroid disease, in particular, overt hypothyroidism, was seen to be of greater significance than thyroid autoimmunity for the risk of pregnancy loss among pregnant women with a prior history of miscarriage and positive anti-TPO.

## Data Availability

The data set generated and/or analyzed during this research are available from the corresponding author upon reasonable request.

## Abbreviations

*TSH*: thyroid-stimulating hormone
*FT3*: free triiodothyronine
*FT4*: free tetraiodothyronine
*anti-TPO*: anti-thyroid peroxidase
*anti-TG*: antithyroglobulin
*ATA*: American Thyroid Association
*NMIS*: number of miscarriages
*CV*: coefficients of variation
*OR*: odds ratios
*COR*: crude odds ratios
*CI*: confidence interval
